# *Candida* meningitis in neurosurgical patients: a single-institute study of nine cases over 7 years

**DOI:** 10.1017/S0950268820001144

**Published:** 2020-05-22

**Authors:** Mantao Chen, Chongchao Chen, Qing Yang, Renya Zhan

**Affiliations:** 1 Division of Neurosurgery, First Affiliated Hospital, School of Medicine, Zhejiang University, Hangzhou 310003, China; 2 Division of Clinical Laboratory, First Affiliated Hospital, School of Medicine, Zhejiang University, Hangzhou 310003, China

**Keywords:** *Candida*, meningitis, neurosurgery, risk factor, therapy

## Abstract

*Candida* meningitis in neurosurgical patients is relatively unusual but is associated with a high mortality rate. We present our experience with this infection and discuss the clinical characteristics, treatment options and outcomes. We retrospectively reviewed neurosurgical patients with multiple positive cerebrospinal fluid (CSF) culture results in our hospital from January 2013 to December 2019. Nine patients were available for review according to our inclusion and exclusion criteria. Four species of *Candida* were isolated from the CSF samples and *Candida albicans* accounted for half of all infections. Eight infections were associated with ventricle peritoneal shunt, lumbar cistern peritoneal shunt or external ventricular drain. All of these foreign intracranial materials were removed or changed and all the patients received antifungal treatment, including fluconazole and/or voriconazole. It is associated with severe long-term outcomes in survivors and a mortality rate that reaches 11.1%. Prior treatments with broad-spectrum and high-grade antibiotics and anaemia are possible risk factors for *Candida* meningitis. We advise that foreign intracranial material should be removed or changed as early as possible and the timing of re-shunt operation can be 1 month after control of *Candida* meningitis has been achieved, with several negative CSF culture results.

## Introduction

While it is estimated that 1.5 million fungal species exist, only approximately 70 000 have been formally described. Among the described species, 300 may be pathogenic in humans and only 10–15% of this influence the central nervous system (CNS) [1, 2]. Fungal CNS infections can be broadly divided into those that infect a healthy host (*Cryptococcus*, *Coccidioides*, *Histoplasma*, *Blastomyces*, *Sporothrix* spp.) and those that cause opportunistic infections in an immunocompromised host (*Candida*, *Aspergillus*, *Zygomycetes*, *Trichosporon* spp.) [1, 3]. The incidence of fungal infections of the CNS has been increasing. CNS fungal infections can present as meningitis, meningoencephalitis, brain abscesses, or stroke syndrome due to vascular invasion [4, 5]. Based on the hospital discharge data, fungal infections accounted for 2.7% of cases of meningoencephalitis in the USA between 2011 and 2014 [6].

Greater numbers of fungal infections are observed among patients belonging to high-risk groups, such as HIV-infected persons, AIDS patients, transplant recipients and immunosuppressed patients treated with chemotherapeutics or corticosteroids, as well as among those suffering from haematological disorders and in patients with chronic diseases [7–10]. Different patient populations are at risk for different species of fungal infections of the CNS [11]. In addition to immunosuppressed individuals, neurosurgical patients are also at risk for fungal infections of the CNS. Although rare, *Candida* spp. and *Cryptococcus* spp. infections have been reported as well as ventriculo-peritoneal shunt infections caused by *H. capsulatum* and *Coccidioides immitis* [4, 12, 13]. *Candida* meningitis in neurosurgical patients has rarely been described [14–16]. When amphotericin B (AmB) combined with or without flucytosine and fluconazole was used as antifungal therapy, the mortality rate of *Candida* meningitis in neurosurgical patients was 11%, 33% and 27%, respectively [14–16]. The emerging importance of *Candida* as a nosocomial pathogen prompted us to review CNS infections caused by *Candida* spp. following neurosurgery in our institution and to examine the clinical characteristics, treatments and outcomes in this cohort of patients.

## Material and methods

We retrospectively reviewed all the culture records from cerebrospinal fluid (CSF) in our hospital from January 2013 to December 2019. Patients with neurological diseases, who needed neurosurgical intervention, with clinical symptoms of CNS infection and positivity of at least two CSF cultures for *Candida* species were included in the study. Patients without signs and symptoms suggestive for meningitis and with meningitis from fungi other than *Candida* were excluded. We reviewed the basic information of each patient, including age, sex, brain diseases, neurosurgical history, antecedent infections and antibiotics. In addition, we collected the risk factors for the development of CNS candidiasis, such as diabetes, immunosuppression, albumin (normal arrange 35–55 g/l) and haemoglobin (normal arrange 131–172 g/l). Clinical data of antifungal therapy and outcomes were collected for each patient in a specific file. Patients without a regular clinical follow-up were called by phone. We also followed up the re-shunt in patients with hydrocephalus. The follow-up duration was 6 months after the last candidiasis episode. Continuous variables were reported as the median and range, while categorical variables were reported as frequencies and percentages. Data analysis was performed in Excel 16.0 (Microsoft Corporation, Redmond, WA, USA).

## Results

We collected 1348 positive culture samples of CSF in our institution in the recent 7-year study period and 70 samples were fungal infection. There were 24 patients with 54 CSF culture samples for *Candida*. Other 16 fungus cultures were *Cryptococcus*, *Rhodotorula mucilaginosa* and *Aspergillus*. In the series of 24 patients with *Candida* in CSF cultures, two patients had no history of neurosurgery and seven patients had no signs and symptoms of meningitis. Four patients were considered as bacterial meningitis and one patient as *Nocardia* meningitis without antifungal therapy. One patient with once *Candida* culture was treated with fluconazole for 3 days and transferred to a local hospital. Eventually, nine patients were available for further review according to our inclusion and exclusion criteria. The clinical data of these patients are listed in Table 1.

**Table 1.** Demographic data, clinical characteristics and outcome in nine cases of Candida meningitis

Patient no.	Age/Sex	Brain disease	Neurosurgical history	History of other diseases	Glucocorticoid	Antecedent infection	Antibiotics	Species	Disseminated candidiasis	Albumin(g/L)	Haemoglobin(g/L)	Treatment	Antifungal agent	Outcome
1	48/M	Trauma	Haematoma clearance, VP, draining of subdural effusion	Hypertension	No	Pulmonary	Sulperazone	*Candida parapsilosis, Candida famata*	None	40.9	85	Shunt removement, external ventricular drainage, lumbar drainage	Fluconazole, Voriconazole	Survived
2	72/M	Glioma	Tumour resection	Hypertension	Yes	Brain	Ceftazidime, vancomycin	*Candida parapsilosis*	None	34	126	Lumbar drainage	Fluconazole	Survived
3	7/M	Trauma	Haematoma clearance, CSF leakage repair, external ventricular drainage, lumbar drainage	None	NK	Brain, pulmonary	NK	*Candida albicans, Candida famata*	None	39.5	134	Lumbar puncture	Fluconazole	Survived
4	70/M	Trauma	Haematoma clearance, lumbar drainage	Hypertension	No	Brain, pulmonary	Linezolid, meropenem	*Candida albicans*	None	37.1	77	Lumbar drainage	Fluconazole,Voriconazole	Death
5	51/M	Trauma	Haematoma clearance, lumbar drainage	None	Yes	Brain, pulmonary	Linezolid, meropenem	*Candida albicans*	Pulmonary (Candida albicans, Candida glabrate)	36.3	79	Lumbar drainage, craniotomy for infection clearance and CSF leakage repair	Fluconazole	Survived
6	38/M	Trauma	Haematoma clearance, CSF leakage repair, lumbar drainage	None	Yes	Brain, pulmonary	Linezolid, meropenem, tigecycline	*Candida albicans*	None	37.2	60	Lumbar drainage	Fluconazole	Survived
7	67/M	Trauma	Haematoma clearance, VP	Hypertension	No	Brain, pulmonary	Linezolid, meropenem,	*Candida parapsilosis*	Pulmonary (Candida albicans)	35.6	87	Shunt removement, lumbar drainage	Fluconazole, Voriconazole	Survived
8	46/F	Trauma	Haematoma clearance, LP	Diabetes	No	Brain, pulmonary, urinary	Meropenem, ciprofloxacin	*Candida albicans*	None	38.3	143	Shunt removement, external ventricular drainage, lumbar drainage	Fluconazole	Survived
9	73/M	Heamorrhage	Haematoma clearance, debridement, external ventricular drainage	Hypertension	No	Brain, pulmonary	Linezolid, meropenem,	*Candida glabrata*	None	32.7	86	Lumbar drainage	Voriconazole	Survived

NK, not known.

In our series, seven cases of brain trauma and one case of cerebellar haemorrhage underwent emergency craniotomy for the clearance of intracranial haematoma, while only one glioma patient underwent elective surgery for intracranial tumour resection. All the patients suffered postoperative infections in the brain, pulmonary system, or urinary system and all of them received one or multiple classes of antibiotics. In our study, four species of *Candida* were isolated from the CSF samples and *Candida albicans* accounted for half. Eight infections were associated with foreign intracranial materials: two with a ventricle peritoneal shunt (VPS), one with a lumbar cistern peritoneal shunt (LPS), one with an external ventricular drain (EVD) and four with a lumbar drain. All foreign intracranial materials were removed or changed and all patients received antifungal agents, including fluconazole and/or voriconazole. Only one patient died after receiving invasive treatment and antifungal agents, with a mortality of 11.1%. In our series, three patients (patient 1, 5 and 8) received the re-shunt for hydrocephalus. Both patient 1 and 8 underwent operations to place shunts operation 1 month after the control of *Candida* meningitis was achieved. Meanwhile, six and five negative CSF cultures were observed, respectively. The follow-up of these two patients were favourable. However, patient 5, who was placed an LPS 2 weeks after the control of *Candida* meningitis with three negative cultures, suffered the infection of *Candida* again. Through removing of the LPS and standard therapy with fluconazole, this patient received the operation of VPS 1 month after the control of *Candida* meningitis with three negative CSF results and cultures.

Our study also collected data of other risk factors for the development of CNS candidiasis. Only one patient had diabetes and three received the therapy of glucocorticoid in the recent 1 month before CNS candidiasis. The median of malnutrition indicator albumin in our series was 37.1 g/l and only two patients were lower than the normal level. Meanwhile, 77.8% of patients suffered from anaemia and six patients reached a moderate level, with a median haemoglobin value of 86 g/l.

## Discussion

The pathogenesis of fungal CNS infections has not been fully described. However, the penetration of fungi across the blood-brain barrier (BBB) is the most probable mechanism responsible for *Candida* meningitis. Three mechanisms by which pathogens cross the BBB have been described: transcellular migration, paracellular migration and the Trojan horse mechanism [17, 18]. The BBB is formed by brain endothelial cells, which are connected by tight junctions and surrounded by astrocyte foot processes that maintain the integrity of the BBB. Fungal cells can be trapped by vascular constriction with possible sensing and signalling of both cell types. Fungal cells can be internalised within a host cell (Trojan horse) that makes contact with the endothelium, arrest and generates sensing and signalling of all three cell types. This is followed by transmigration that could be via the trans- or paracellular mechanism. Immune and inflammatory cells are recruited to the vascular or extracellular compartment as part of the host defence and inflammatory response [19]. In our opinion, the BBB is directly disrupted by neurosurgery and the reduction in immunity after neurosurgery increases the permeability of the BBB. These factors facilitate the penetration of fungi into the brain and lead to fungal infection of the CNS. In addition, the colonisation of a foreign device may also represent a reason for the infection when the device is inside brain ventricles.

Due to the development of nonculture-based diagnostic techniques, which allow more sensitive and rapid identification of fungi, the incidence of fungal infections of the CNS has been increasing. However, *Candida* meningitis in neurosurgical patients has rarely been described. It is important to note that *Candida albicans* is commonly isolated from both immunocompromised and immunocompetent patients, but its prevalence has begun to decrease over the past decade, while the frequency of detection of non-*C. albicans* species has gradually increased. It should be noted that the incidence of *Candida* spp. has increased from 6 to 17% in fungal infections of the CNS [9]. Before the 21th century, Nguyen and Yu (1995) reviewed three of their own cases of neurosurgery-related *Candida* meningitis and 15 cases previously reported in the English-language literature. They found that most patients with *Candida* meningitis had recently received antibacterial agents and it is notable that 50% of patients suffered from antecedent bacterial meningitis. In addition, the overall mortality rate in those 18 patients was 11% [14]. Another retrospective study by Geers and Gordon (1999) reviewed 48 *Candida* isolates cultured from CSF samples from 21 post-neurosurgery patients. *C. albicans* was isolated from 48% of the patients. Bacterial meningitis appears to be associated with the development of *Candida* meningitis and preceded *Candida* meningitis in 63% of cases. The overall mortality rate associated with *Candida* meningitis in adult patients following neurosurgery was 27%. The mortality rate among our post-neurosurgery patients with clinically significant *Candida* meningitis (excluding those not treated with antifungal agents) was 33% [15]. After the 21th century, O'Brien *et al*. (2011) reported 11 cases of *Candida* CSF infection after neurosurgery over a 12-year period. In total, 73% of the isolates were *C. albicans* and 73% of the patients had antecedent bacterial meningitis. All infections were associated with foreign intracranial material, including EVD, VPS, lumbar drain and Gliadel wafers, with a mortality rate of 27% [16]. In our study, all nine patients contracted postoperative infections and eight patients had an intracranial bacterial infection before developing *Candida* meningitis. These patients received multiple classes of antibiotics due to multidrug-resistant bacteria, multiple bacterial infections and multisystem infections. These broad-spectrum and high-grade antibiotics prompted the development of fungal infections. In addition, only one patient died after receiving invasive treatment and antifungal agents. The mortality rate was 11.1%, which was lower than that in the previous studies.

In the previous studies, the post-craniotomy intracranial *Candida* infection was associated with prior use of antibacterial agents and foreign intracranial materials such as VPS, LPS, EVD and Gliadel wafers [14–16, 20]. There was the same result in our study. The susceptible populations for *Candida* spp. infection include patients with neutropenia, solid organ transplants, corticosteroids, autoimmune disorders and neurosurgery [11]. Besides, diabetes mellitus is a risk factor of intracranial infection for neurosurgery [21]. Our study did not get the same results for corticosteroids and diabetes mellitus due to the small populations. Dunne *et al*. (2002) found perioperative anaemia was an independent risk factor for infection and mortality in surgery [22]. We found 77.8% of patients suffered from anaemia, suggesting anaemia as a possible association with *Candida* infection.

Due to the low morbidity of *Candida* meningitis, no randomised controlled trials have been performed to evaluate the most appropriate treatment; only single case reports and small series exist. In our study, all patients were treated with fluconazole and/or voriconazole. A total of 88.9% of patients survived after treatment. Most reports have demonstrated the use of AmB with or without flucytosine [15]. AmB is a polyene antifungal agent with activity against all *Candida* spp. of clinical importance; evidence supports the use of AmB for CNS infections [23]. 5-Flucytosine possesses excellent penetration into the CSF and has activity against most clinically important fungi, but its toxicity precludes its widespread use. Human studies have also demonstrated the excellent penetration of fluconazole into the CNS; however, intrinsic resistance to this azole is present in certain *Candida* species, e.g. *Candida glabrata*, which limits its usefulness as a first-line agent [24]. According to the clinical practice guidelines for the management of candidiasis by the Infectious Diseases Society of America updated in 2016, liposomal AmB (AmB, 5 mg/kg daily) with or without oral flucytosine (25 mg/kg 4 times daily) is strongly recommended as the initial therapy. However, the optimal length of initial therapy has not been studied. Fluconazole has proven useful as a step-down therapy at a dosage of 400–800 mg (6–12 mg/kg) daily due to its excellent levels in the CSF and brain tissue. The indication for the cessation of therapy is when all signs, symptoms and CSF and radiological abnormalities have resolved [25].

Not only case reports but also the small series strongly recommended removing implanted devices from patients with *Candida* infections [14, 16, 20], guidelines suggest that infected CNS devices, including ventriculostomy drains, shunts, stimulators, prosthetic reconstructive devices and biopolymer wafers that deliver chemotherapy, should be removed if possible [25]. All of these foreign intracranial materials were removed or changed in our study. However, three patients with VPS or LPS received antifungal agents, repeated lumbar puncture and even lumber drainage or external ventricular drainage, in the beginning to attempt to retain the shunt. The failure of therapy prompted us to remove the shunt. Therefore, we advise removing or changing foreign intracranial materials as early as possible.

The timing of replacing the shunt in patients with hydrocephalus is not clear yet. Both patient 1 and 8, achieved success in the re-shunt 1 month after the control of *Candida* meningitis with more than five negative CSF cultures. While patient 5 failed in the LPS operation 2 weeks after the control of *Candida* meningitis with three negative culture results. This patient received the operation of VPS 1 month after the control of *Candida* meningitis with three negative CSF results and cultures. Hence, we advise that the operation to replace a removed shunt can take place 1 month after controlling the *Candida* meningitis, with several negative CSF culture results. Due to the lack of cases and clinical therapy experiences, the timing of replacing the shunt in patients with hydrocephalus requires further research.

## Conclusions

The mortality of *Candida* meningitis in neurosurgical patients is high. An antecedent infection, especially bacterial meningitis, with broad-spectrum antibiotics and high-grade antibiotics and anaemia are possible high-risk factors for *Candida* meningitis. Early diagnosis with CSF culture and early treatment with antifungal agents is beneficial for patients, while foreign intracranial material, such as VPS, LPS and EVD, should be removed or changed as early as possible. We advise that the operation to replace the removed shunt can take place 1 month after controlling the *Candida* meningitis, with several negative CSF culture results.
